# OR5-002 – In vitro studies in Schnitzler’s syndrome

**DOI:** 10.1186/1546-0096-11-S1-A95

**Published:** 2013-11-08

**Authors:** HD De Koning, J Schalkwijk, J Jongekrijg, IM van Vlijmen-Willems, D Rodijk-Olthuis, M Stoffels, JW van der Meer, PL Zeeuwen, A Simon

**Affiliations:** 1 Dermatology, Nijmegen, the Netherlands; 2 Radboud University Nijmegen Medical Centre, Nijmegen, the Netherlands; 3 Internal Medicine, Radboud University Nijmegen Medical Centre, Nijmegen, the Netherlands

## Introduction

Schnitzler’s syndrome (SchS) is an autoinflammatory disorder, characterized by chronic urticaria, fever, gammopathy and bone pain. The pathophysiology is unknown, but the effectiveness of interleukin-1 (IL-1) inhibition provides a clue.

## Objectives

Our aim was to study the effect of IL-1β inhibition on inflammatory responses *in vivo* and *ex vivo* during a trial of the long-acting anti-IL-1β antibody canakinumab in SchS.

## Methods

Eight patients with SchS received monthly injections with 150mg canakinumab s.c. for six months. Blood was drawn at several time points for measurement of inflammation markers and isolation of peripheral blood mononuclear cells (PBMCs), which were stimulated with lipopolysaccharide (LPS). Skin biopsies of urticae and clinically uninvolved skin were taken for mRNA, histology and keratinocyte cultures. Submerged keratinocytes were stimulated with several cytokines and patient and control serum. All data were compared to results of healthy controls.

## Results

IL-1β inhibition was highly effective in SchS. IL-6 protein concentration in lysates of freshly isolated PBMCs correlated with disease activity. Stimulation of PBMCs with 0,1 ng/ml LPS induced more IL-6 and IL-1β production in SchS PBMCs than in controls. In lesional epidermis, mRNA and protein expression levels of several antimicrobial proteins were elevated. In primary human keratinocytes, poly:IC, IL-1β, IL-17 and interferon gamma induced mRNA expression levels of several antimicrobial proteins and cytokines to a similar extent in patient and control cells.

## Conclusion

Clinical efficacy of IL-1β inhibition in patients with SchS is associated with *in vivo* and *ex vivo* suppression of inflammation. Our data underscore that IL-1β plays a pivotal role in this disease. Also, we show strong upregulation of antimicrobial proteins in the epidermis of these neutrophilic urticaria, and that these proteins can be induced in primary human keratinocytes by IL-1β.

## Disclosure of interest

None declared

